# Supplementary figures and images for: Spatiotemporal coordination of reovirus peripheral core replication to perinuclear whole virus assembly
Source: PLoS Pathog. 2025 Sep 2;21(9):e1013238. doi: 10.1371/journal.ppat.1013238 (PMC12413085; doi:10.1371/journal.ppat.1013238)

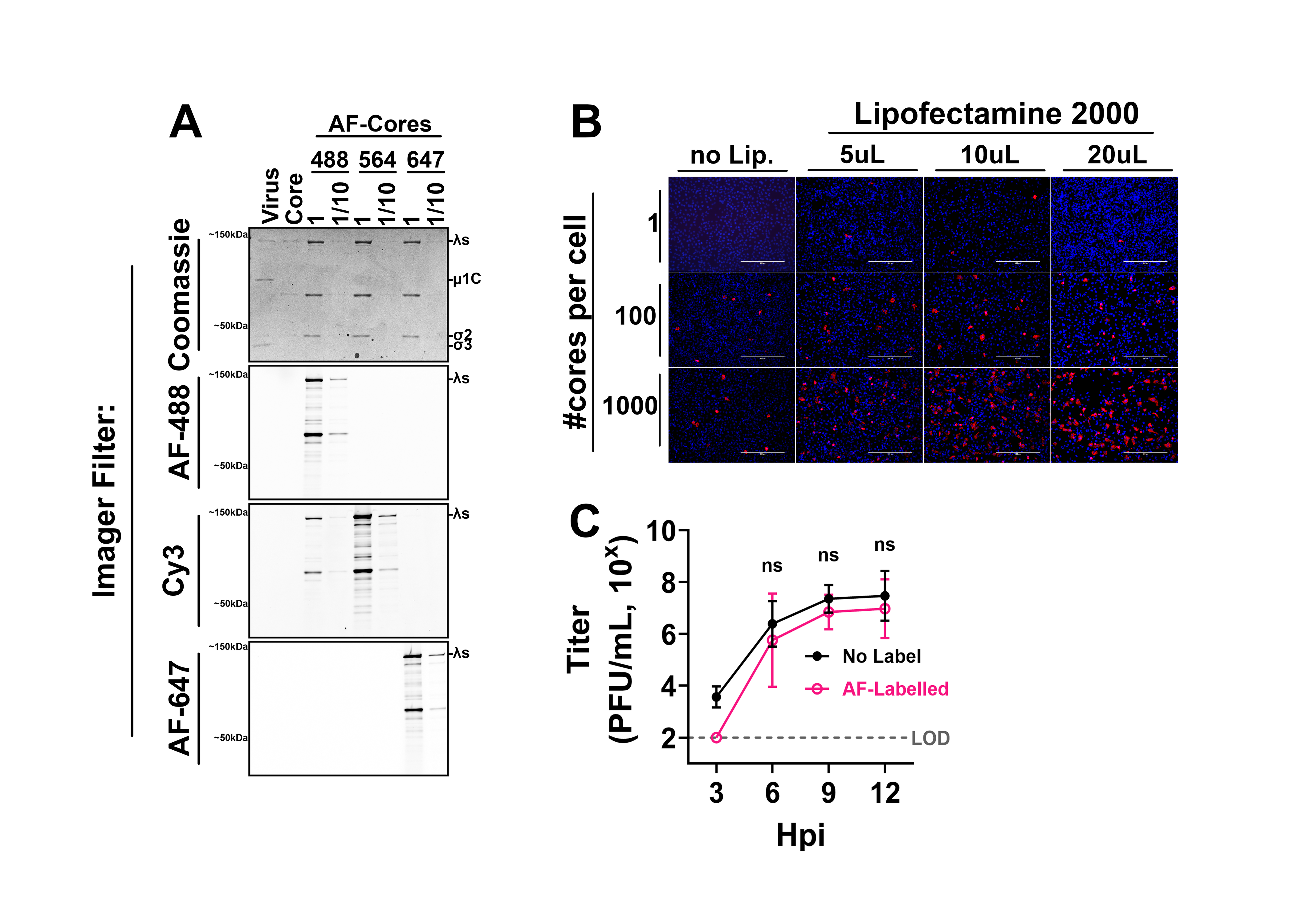

Supplement: S1 Fig — T3DPL virions were digested with chymotrypsin to generate core particles in-vitro. A subset of cores was then labelled with AF-488, -564, or -647 dyes. (A) Whole virus (virus), non-labelled core particles (core) and labelled cores (AF-cores) were subject to SDS-PAGE. The gel was then immediately imaged under AF-488, Cy3, and AF-647 filters using BioRad’s ChemiDoc MP imaging system. Then, the same gel was stained with Coomassie and imaged again for total protein identification. (B) The ratio of non-labelled cores to volume of Lipofectamine 2000 transfection reagent was assessed in H1299 cells. 18 hpt, cells were fixed and immunostained using polyclonal sera from rabbits immunized with reovirus particles (α-Reo, red) and stained with DAPI to visualize nuclei. (C) ~1000 non-labelled (Cores, black line, n = 5) AF-Cores (blue line, n = 3) per cell were transfected into H1299 cells. Lysates were collected every 3 hours for 12 hours and virus production was assessed via plaque assay. Data is graphed as the mean + /- SD at each timepoints. Statistical analysis is reported as two-way ANOVA with Sidak’s multiple comparisons test between the mean of each group at their respective timepoints. ****p < 0.0001, ***p < 0.001, **p < 0.05, ns > 0.05. In vitro core digestions can leave some intact whole virions that produce some background fluorescence when immunostaining for de novo virus proteins in no-lipofectamine conditions. Such whole virions however would not have AF-labelled cores, and the potentially AF-labelled outercapsid proteins would be shed during endocytosis. Nevertheless, we used conditions that provided notable increase in fluorescence in the presence of lipofectamine over the absence of lipofectamine, to skew towards a majority proportion of AF-core-driven infection. (TIF) [file ppat.1013238.s001.tif]

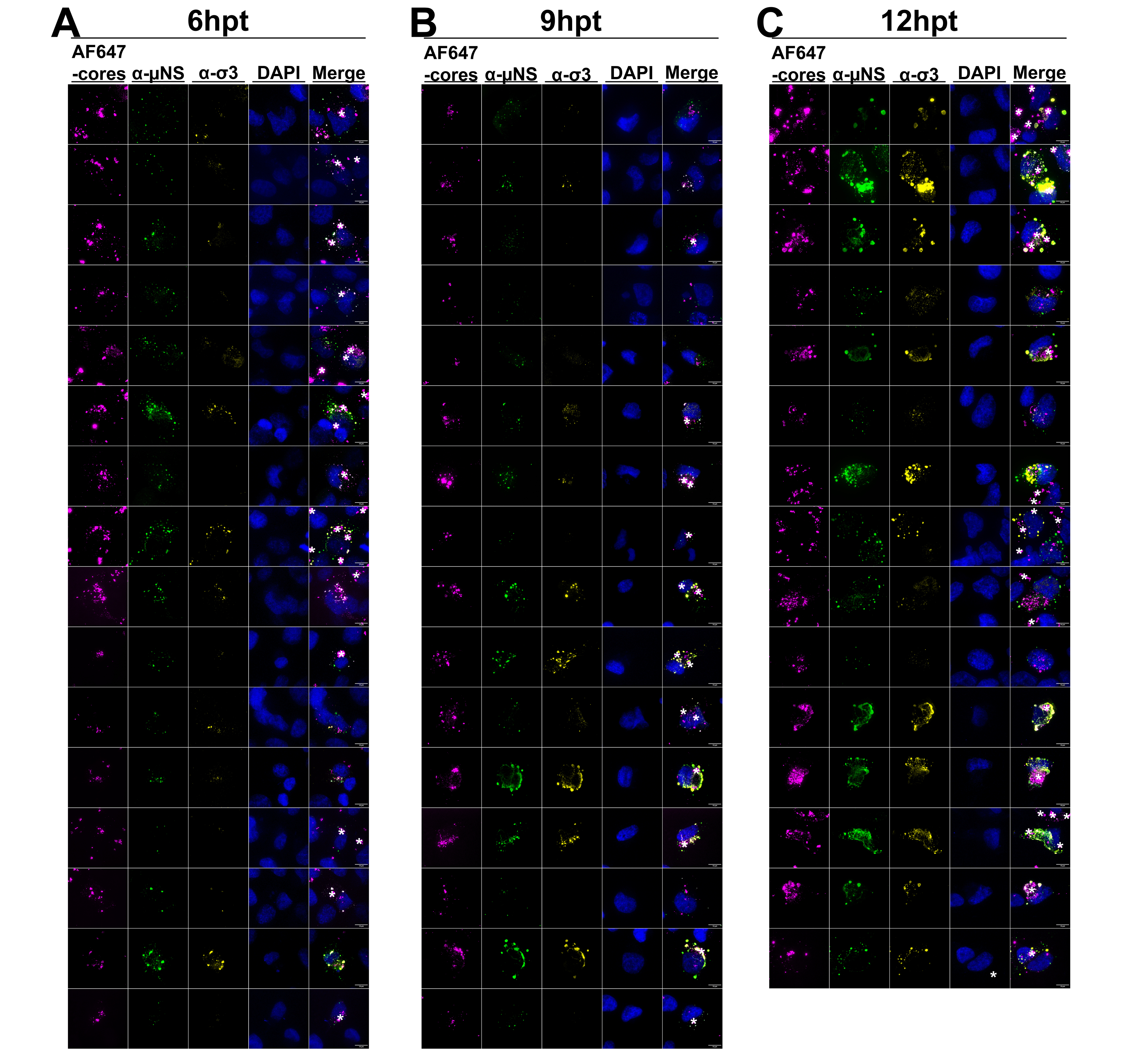

Supplement: S2 Fig — H1299 cells were transfected with ~1000 AF 647-labelled reovirus core particles per cell (magenta). At (A) 6, (B) 9 and (C) 12 hpt cells were fixed and subsequently immunostained with rabbit polyclonal sera generated against μNS (AF 488, green), mouse monoclonal anti-σ3 (10G10, Cy3, yellow) and nuclei were stained with DAPI (blue). All images captured by spinning disk confocal microscopy and are images of compressed Z-stacks. White asterisks indicate aggregates formed by AF-cores. (TIF) [file ppat.1013238.s002.tif]

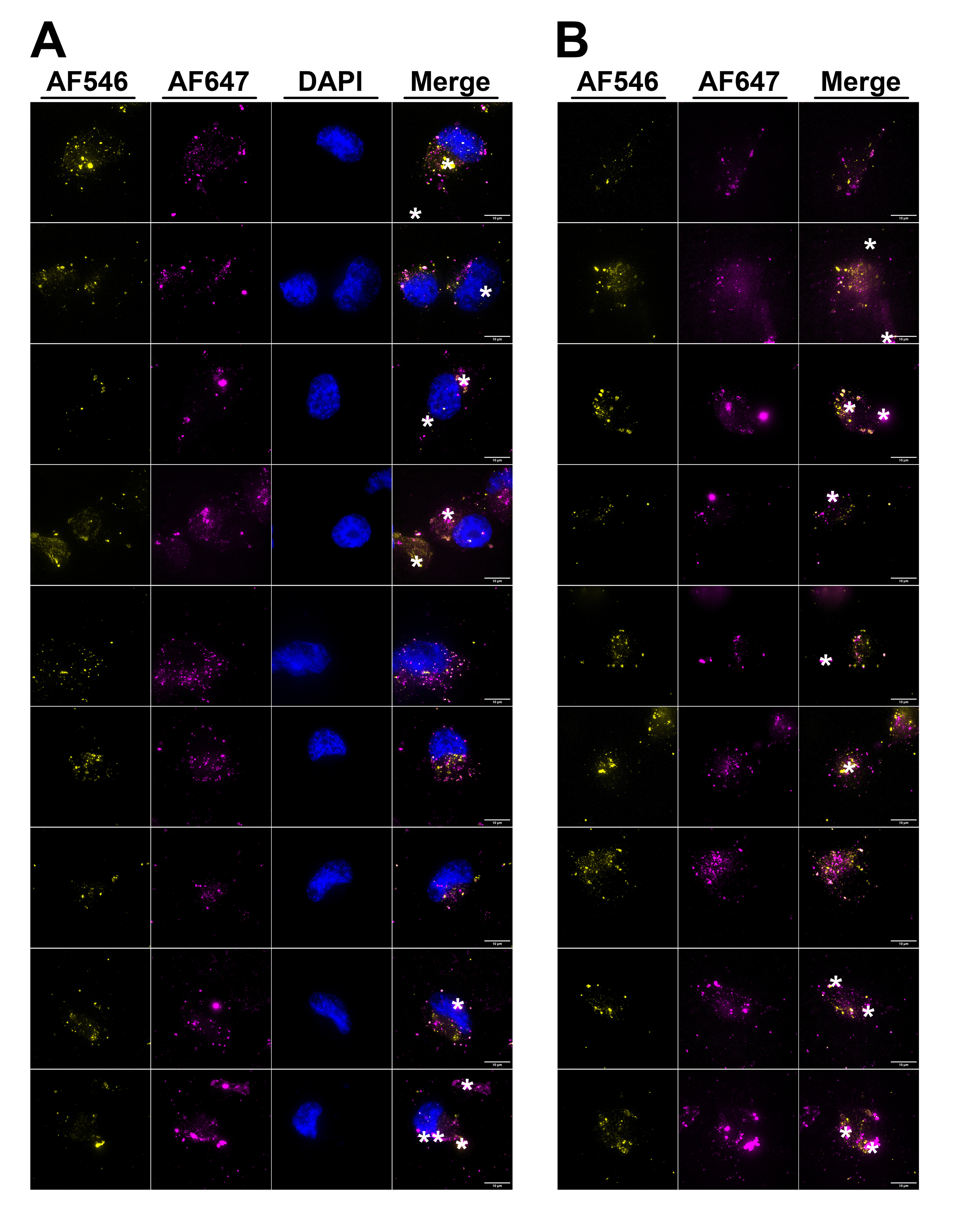

Supplement: S3 Fig — H1299 cells were first transfected with ~1000 AF-564 cores per cell. One hour later, the cells were transfected with ~1000 particles per cell of AF-647 labelled cores. 7 hpt, cells were fixed and processed for immunofluorescence confocal microscopy imaging. Representative images of compressed Z-stacks. White asterisks indicate aggregates formed by AF-cores. (A) Cells were stained with DAPI to visualize nuclei. (B) Cells were not stained with DAPI. (TIF) [file ppat.1013238.s003.tif]

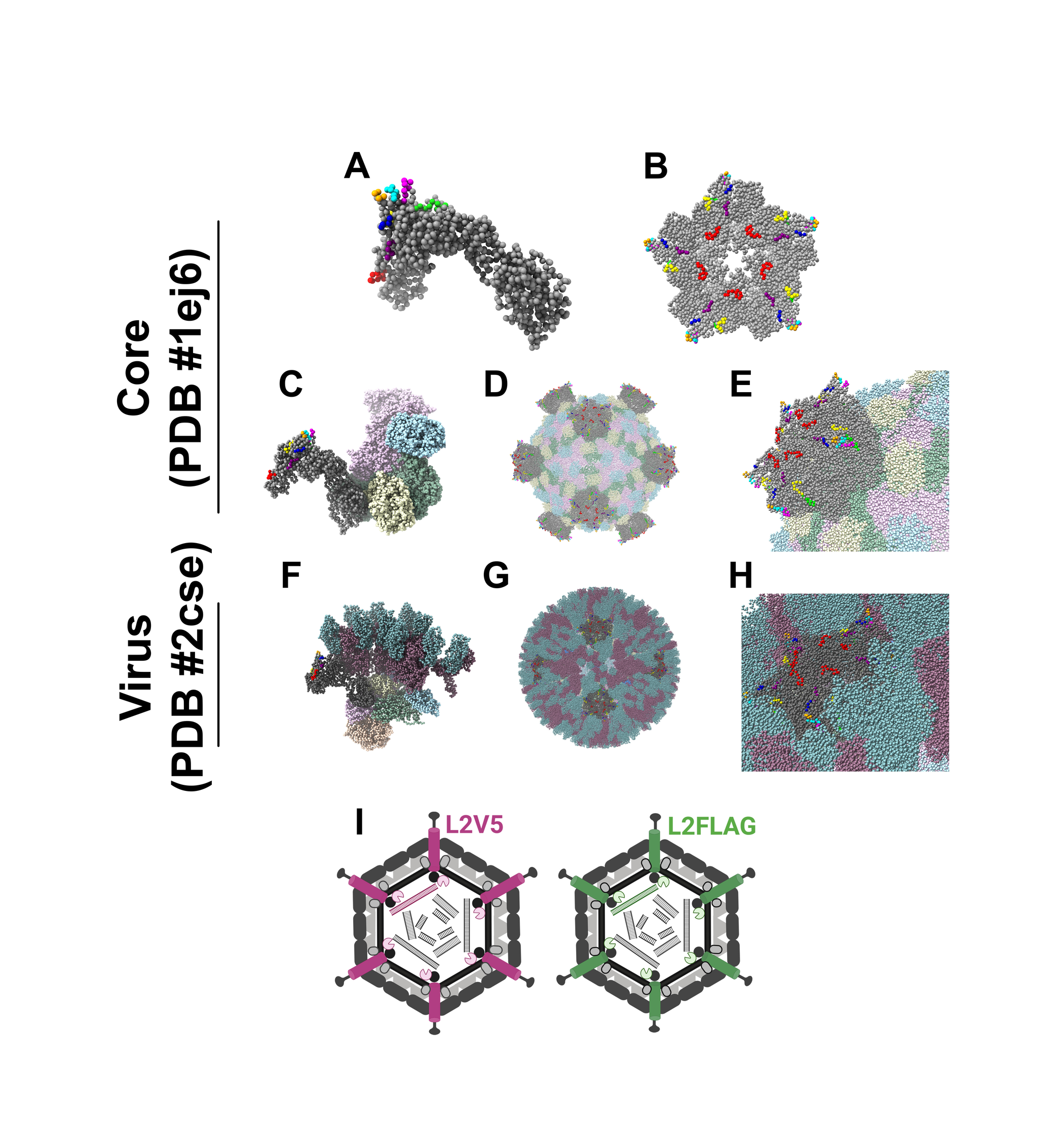

Supplement: S4 Fig — Schematics demonstrating amino acid regions tested for incorporation of a 2xFLAG tag or 1xV5 tag. Colours correlate with those used in Table 1. (A-E) Modeling of λ2 attempted tag regions (structures from PDB accession #1EJ6) in (A) the λ2 monomer, (B) the λ2 pentamer, (C) the asymmetric core unit, (D) the whole assembled core, and (E) a zoomed in pentamer assembled within the core. (F-H) Modeling of the λ2 attempted tag regions (PDB #2CSE) in (F) the whole virion asymmetric unit, (G) the whole assembled virion, and (H) a zoomed in pentamer assembled within the whole virus. (I) Cartoon schematic showing the tagged viruses generated in the reverse genetics system, with tagged proteins colour coded. These are the same icons shown in Figure 3B, created in BioRender. Shmulevitz, M. (2025) https://BioRender.com/3n918ke. (TIF) [file ppat.1013238.s004.tif]

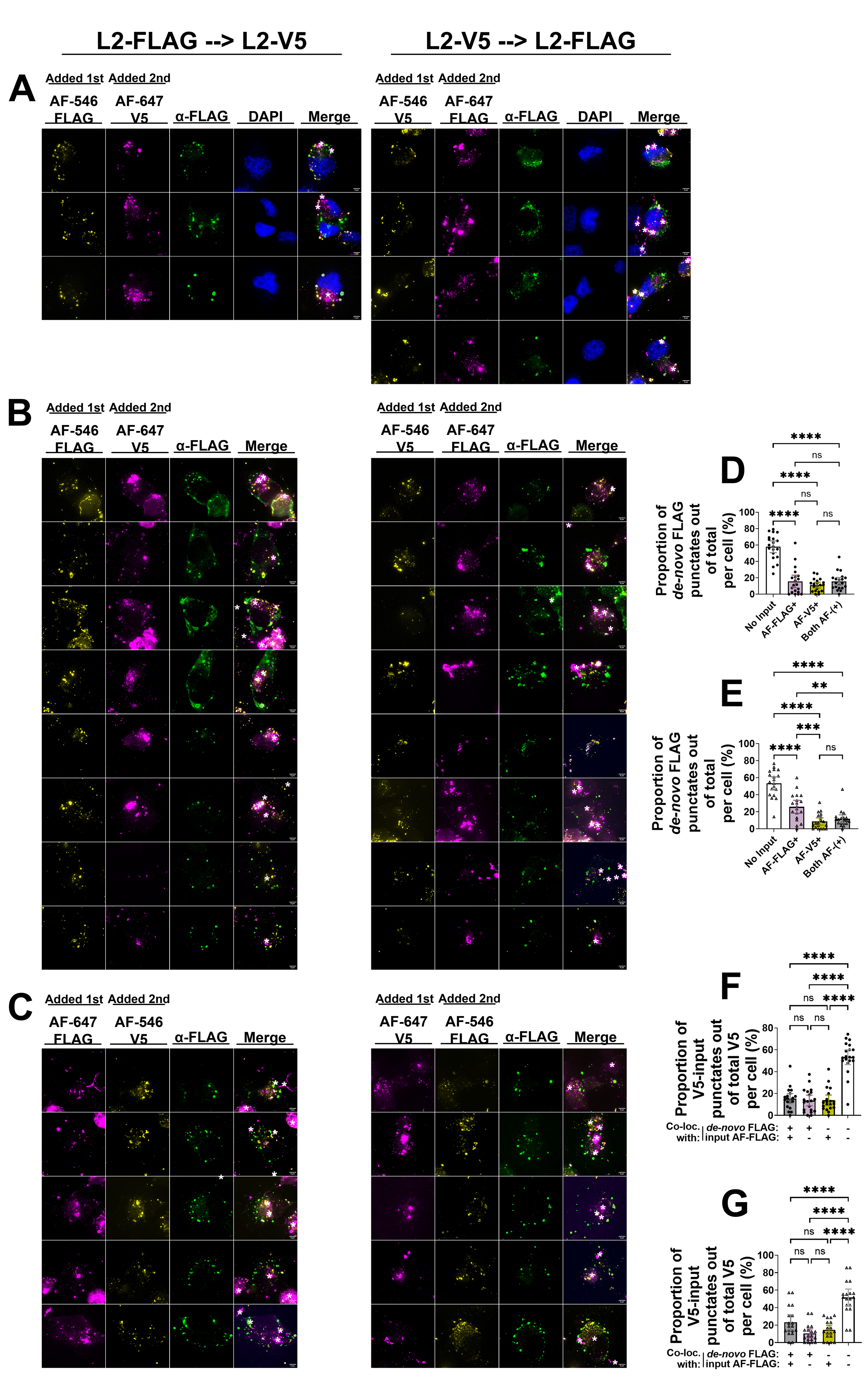

Supplement: S5 Fig — (A-B) H1299 cells were first transfected with ~1000 AF-546 labelled FLAG- or V5-tagged cores per cell. One hour later, the cells were transfected with ~1000 particles per cell of AF-647 labelled FLAG- or V5-tagged cores (opposite tag to the first transfection). 7 hpt, cells were fixed and processed for IF-CM. Representative images of compressed Z-stacks. White asterisks indicate aggregates formed by AF-cores. (A) Cells were immunostained with monoclonal mouse α-FLAG (AF 488, green) and stained with DAPI to visualize nuclei. (B-C) Cells were immunostained with α-FLAG (AF 488, green). (C) H1299 cells were first transfected with ~1000 particles per cell of AF-647 labelled FLAG- or V5-tagged cores. 1 hour later, the cells were transfected with ~1000 particles per cell of AF-546 labelled FLAG- or V5-tagged cores. 7 hpt, cells were fixed and processed for IF-CM. Representative images of compressed Z-stacks. (D and E) Graphs represent the same data displayed in Figure 4C but split to represent (D) data sets from where AF-FLAG particles were transfected first, and (E) where AF-V5 particles were transfected first. Similarly, (F and G) graphs represent the same data displayed in Figure 4D but split to represent (F)) data sets from where AF-FLAG particles were transfected first, and (G) where AF-V5 particles were transfected first. Data is plotted as mean + /- 95% CI. Statistical analysis is reported as ordinary one-way ANOVA between the mean of each column. ****p < 0.0001, ***p < 0.001, **p < 0.05, ns > 0.05. (TIF) [file ppat.1013238.s005.tif]

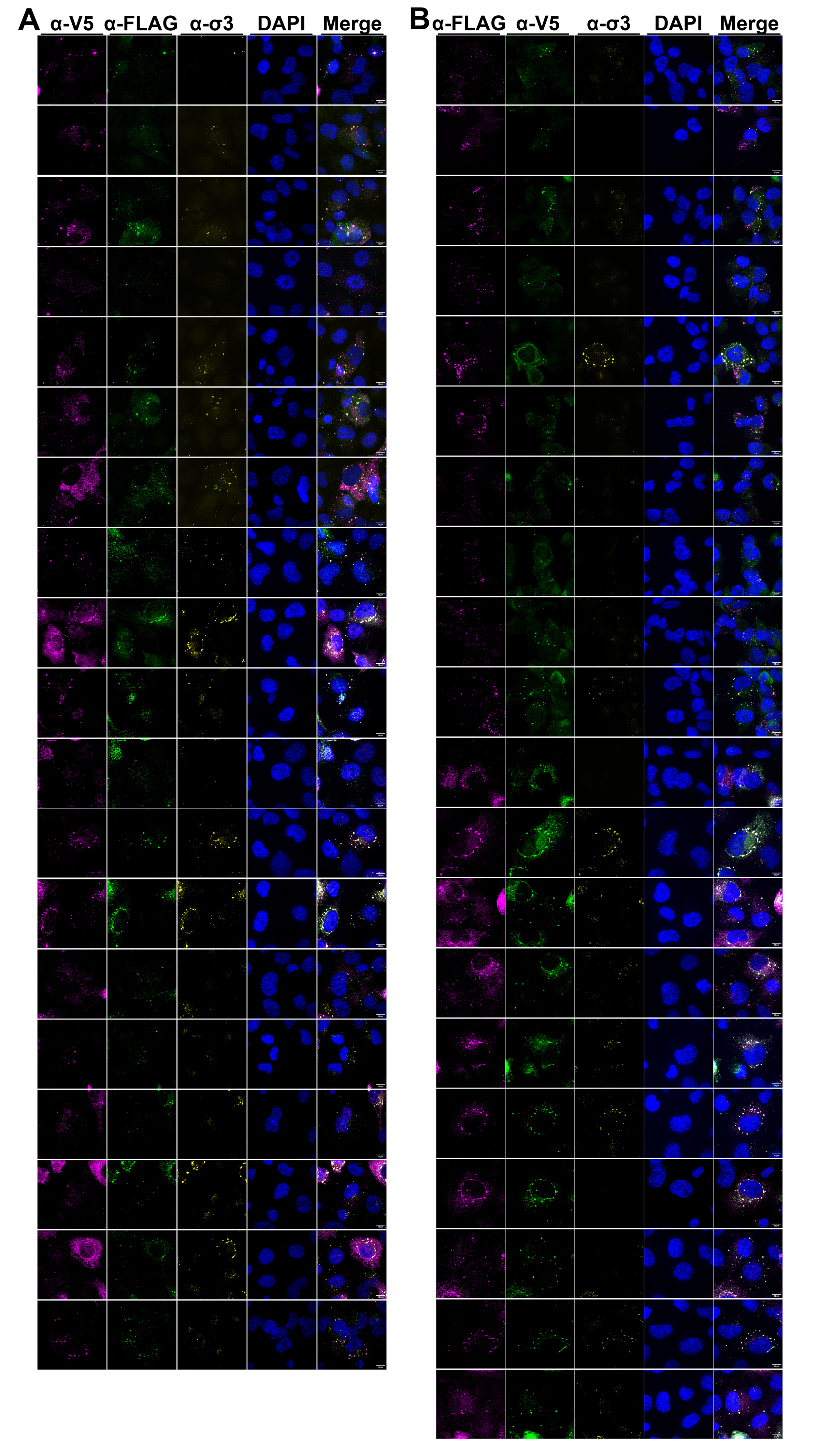

Supplement: S6 Fig — Immunofluorescent images captured via spinning disk confocal microscopy. H1299 cells were co-infected with λ2-FLAG and λ2-V5 at an MOI of 5 each. 8 hpi, cells were fixed and immunostained with (A) monoclonal mouse α-FLAG (AF 488, green), polyclonal rabbit α-V5 (AF 647, magenta) and monoclonal mouse α-σ3 directly conjugated to Alexa Fluor 594 (10C1, yellow) and stained with DAPI for nuclei visualization (blue). (B) The secondary antibodies were swapped from (A), and α-FLAG staining was coupled with AF 647 (magenta) and α-V5 was coupled with AF 488 (green). (TIF) [file ppat.1013238.s006.tif]

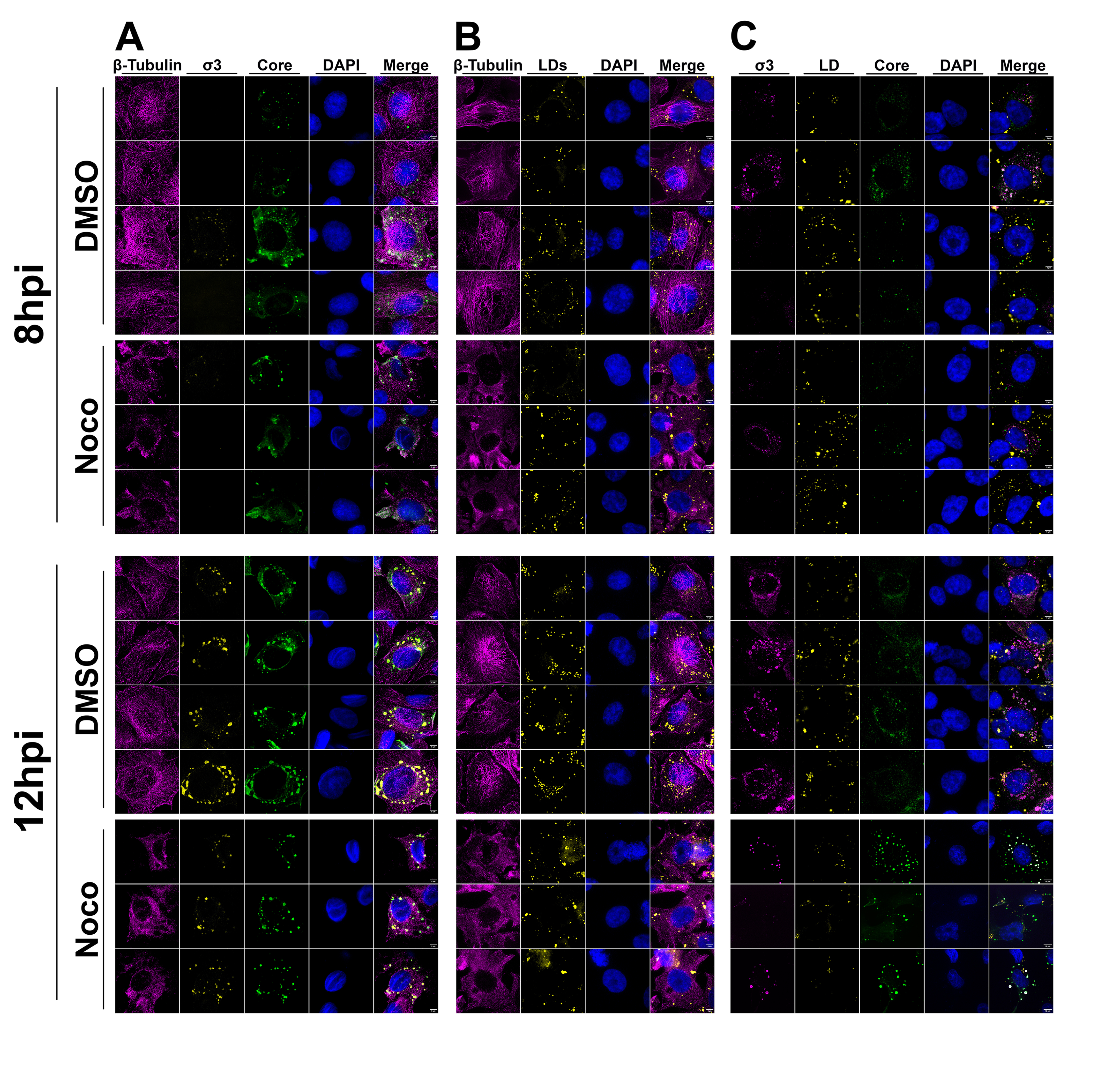

Supplement: S7 Fig — H1299 cells were infected with T3DPL MOI 3. 1 hpi, complete media containing 10μM nocodazole, or an equivalent volume of DMSO (control), was added to the cells. 8 or 12 hpi, cells were fixed and immunostained for immunofluorescence confocal microscopy imaging. Compressed Z-stack images of cells stained with: (A) monoclonal mouse anti-Tubulin (12G10, AF 647, magenta), monoclonal mouse anti-σ3 (10C1, directly conjugated to AF 594, yellow), polyclonal rabbit antibodies raised against reovirus cores (AF 488, green), and DAPI for nuclei visualization (blue). (B) Tubulin (12G10, AF 647, magenta), BODIPY 493/503 for lipid droplet visualization (LDs, yellow), and DAPI for nuclei visualization (blue). (C) Monoclonal mouse anti-σ3 (10G10 followed by AF 647 conjugated secondary antibodies (magenta)), BODIPY 493/503 for lipid droplet visualization (LDs, yellow), and polyclonal rabbit antibodies raised against reovirus cores (AF 488, green). (TIF) [file ppat.1013238.s007.tif]

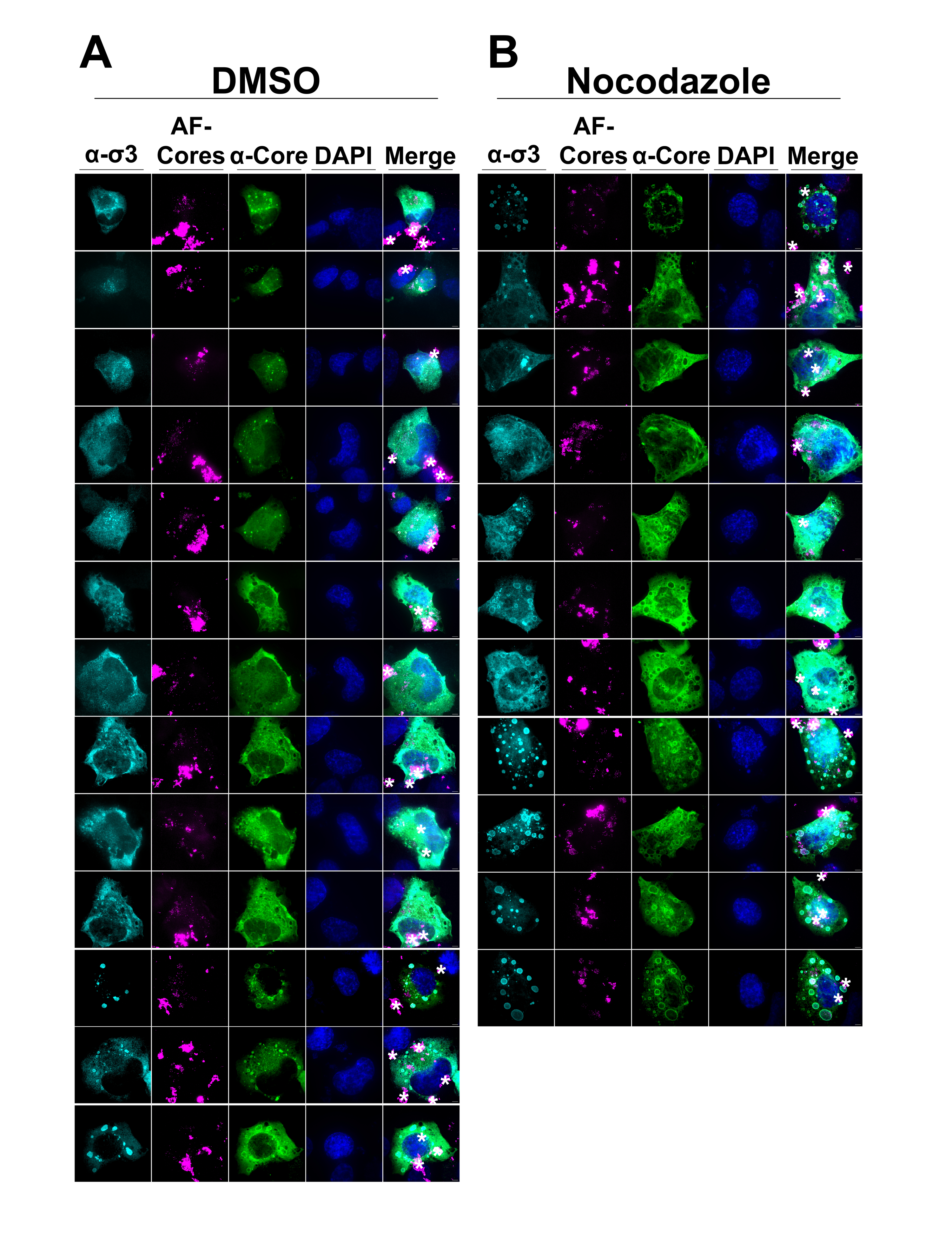

Supplement: S8 Fig — H1299 were transfected with ~1000 AF-546 reovirus core particles per cell (magenta). 1 hpt, cells were treated with (A) DMSO or (B) 10μM nocodazole. 12 hpt, cells were fixed and immunostained with monoclonal mouse antibodies directed against σ3 (10G10, α-σ3, AF 647, cyan) polyclonal rabbit antibodies raised against reovirus cores (α-core, AF 488, green) and DAPI was used to stain nuclei. White asterisks indicate aggregates formed by AF-cores. All images are of compressed Z-stacks captured by IF-CM. (TIF) [file ppat.1013238.s008.tif]

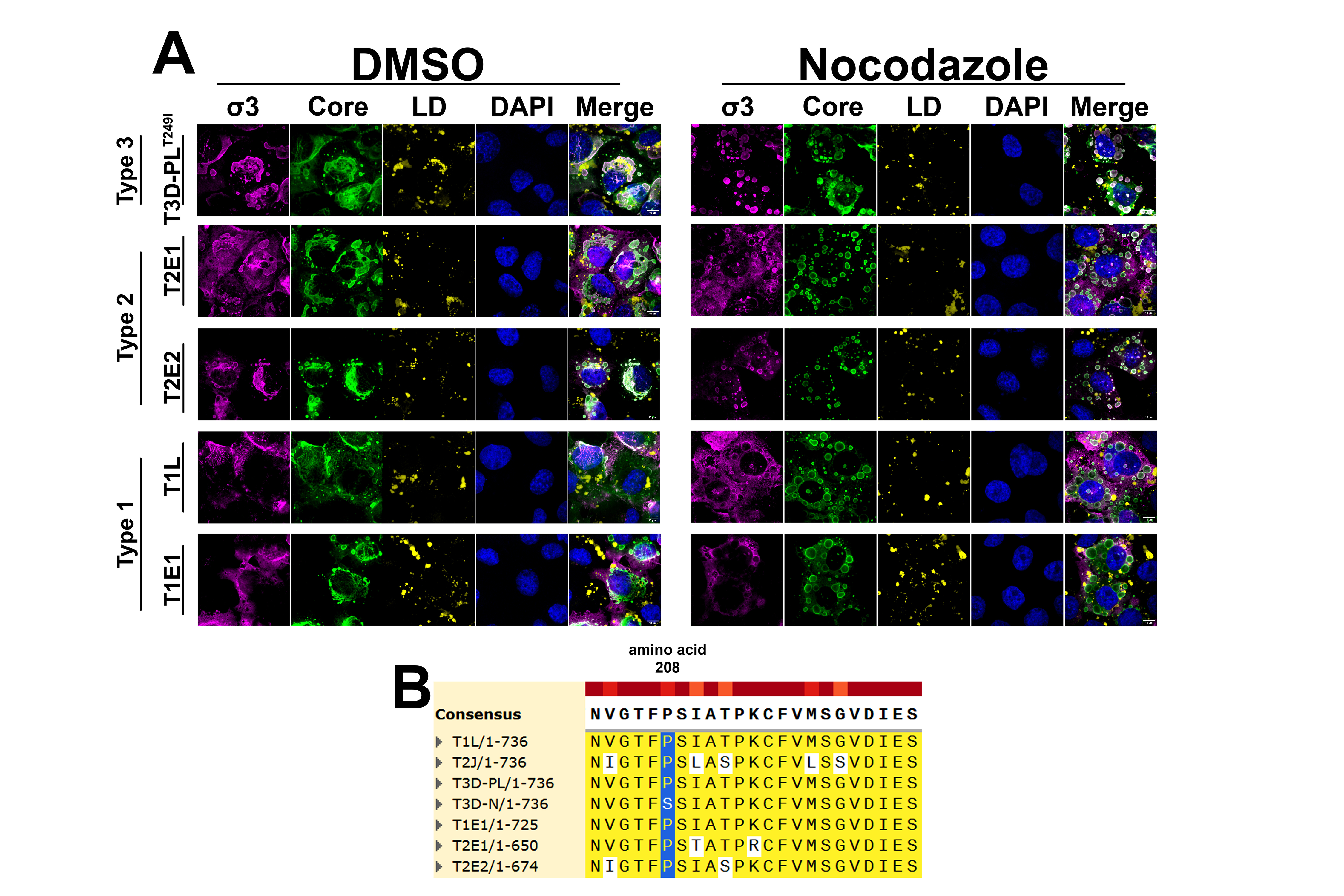

Supplement: S9 Fig — (A) H1299 cells were infected with reovirus ISVP strains Type 3 (T3DS1-T249I), Type 2 (T2E1, T2E2), or Type 1 (T1L, T1E1) to give 80% infection of cells by 12 hpi. 1 hpi, media containing either 10μM nocodazole or an equivalent volume of DMSO were added to the cells. 12 hpi, cells were fixed and immunostained with monoclonal anti-σ3 (10G10, AF 647, magenta) and polyclonal rabbit antibodies raised against reovirus cores (AF 488, green), and stained with BODIPY 493/503 for lipid droplet visualization (LDs, yellow) and DAPI for nuclei visualization (blue). Images are representative immunofluorescence confocal microscopy Z-stacks, of n = 5–10 images per virus type. (B) M1 gene sequences were acquired from NCBI Genbank and aligned using ClustalOmega with default settings. Alignments were then viewed using SnapGene software. Amino acids at position 208 are highlighted in blue. (TIF) [file ppat.1013238.s009.tif]

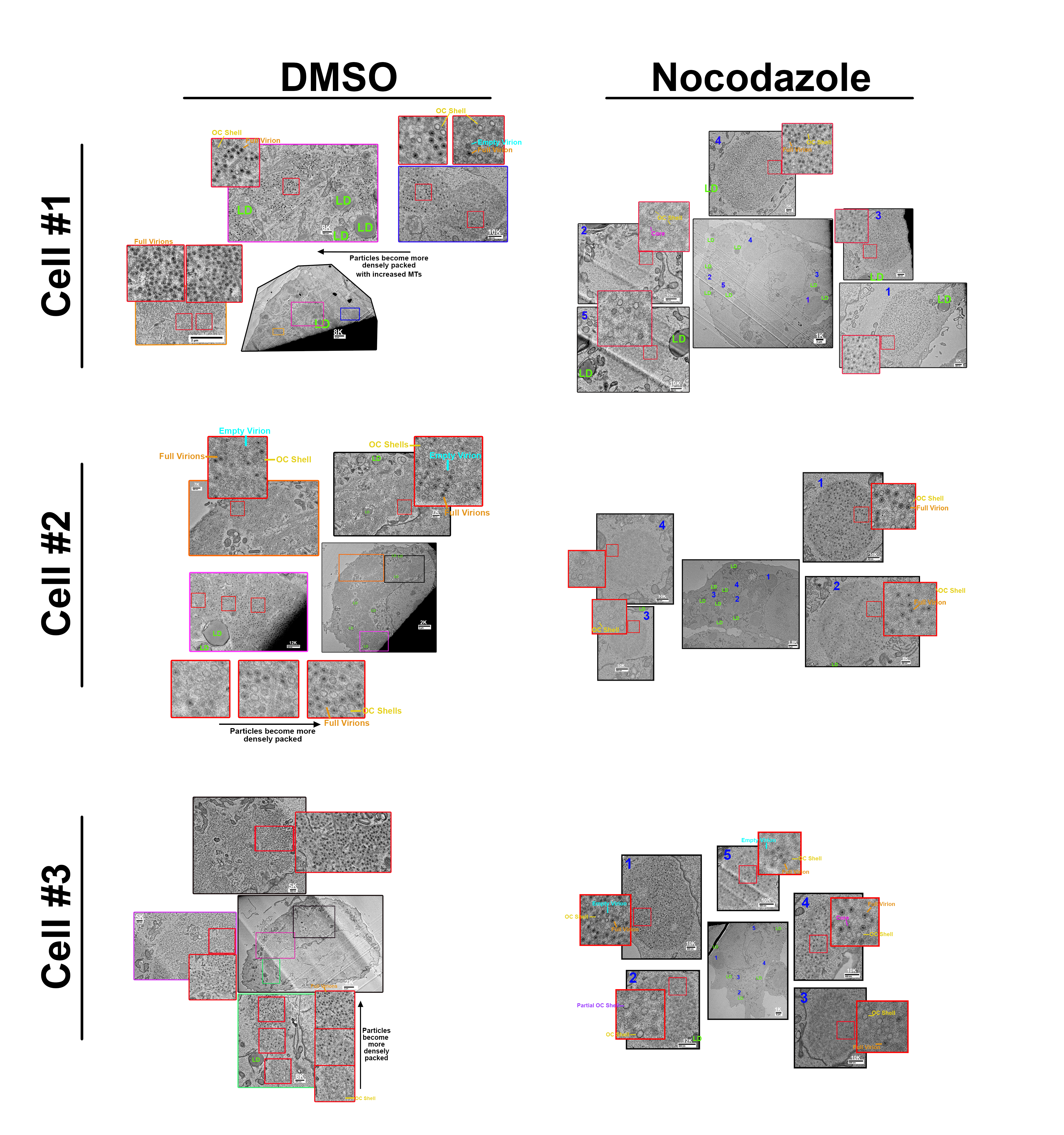

Supplement: S10 Fig — H1299 cells were infected with T3DPL MOI 3. 1 hpi, complete media containing 10μM nocodazole, or an equivalent volume of DMSO was added. Cells were fixed and processed for transmission electron microscopy analysis as described in the methods section at 18 hpi. Select factory regions showcasing the arrangement of cores, full virions, empty virions, and OC shells are shown in the boxed regions. (TIF) [file ppat.1013238.s010.tif]
