# Supplementary material for: Spatiotemporal coordination of reovirus peripheral core replication to perinuclear whole virus assembly
Source: PLoS Pathog. 2025 Sep 2;21(9):e1013238. doi: 10.1371/journal.ppat.1013238 (PMC12413085; doi:10.1371/journal.ppat.1013238)
Supplement: Attachment — Submitted filename: 25.07.27 Letter to editor and reviewers.docx [file ppat.1013238.s012.docx]

July 27, 2025

**RE: PPATHOGENS-D-25-01291 Manuscript revision**

Dear Editors and reviewers,

Thank you for reviewing our manuscript and clearly indicating the necessary revisions. We owe many thanks to the editors and reviewers for their careful evaluation and suggestions. We have now addressed all suggestions with modifications to the manuscript and by inclusion of electron microscopy data.

Sincerely,

Maya Shmulevitz
Canada Research Chair in Molecular Virology and Oncotherapy
Professor
